# Peptide length significantly influences *in vitro *affinity for MHC class II molecules

**DOI:** 10.1186/1745-7580-4-6

**Published:** 2008-11-26

**Authors:** Cathal O'Brien, Darren R Flower, Conleth Feighery

**Affiliations:** 1 Department of Immunology and Institute of Molecular Medicine, St James's Hospital and Trinity College Dublin, Ireland; 2 The Jenner Institute, University of Oxford, Compton, Berkshire, RG20 7NN, UK

## Abstract

**Background:**

Class II Major Histocompatibility Complex (MHC) molecules have an open-ended binding groove which can accommodate peptides of varying lengths. Several studies have demonstrated that peptide flanking residues (PFRs) which lie outside the core binding groove can influence peptide binding and T cell recognition. By using data from the AntiJen database we were able to characterise systematically the influence of PFRs on peptide affinity for MHC class II molecules.

**Results:**

By analysing 1279 peptide elongation events covering 19 distinct HLA alleles it was observed that, in general, peptide elongation resulted in increased MHC class II molecule affinity. It was also possible to determine an optimal peptide length for MHC class II affinity of approximately 18–20 amino acids; elongation of peptides beyond this length resulted in a null or negative effect on affinity.

**Conclusion:**

The observed relationship between peptide length and MHC class II affinity has significant implications for the design of vaccines and the study of the epitopic basis of immunological disease.

## Background

Classical major histocompatibility complex (MHC) molecules are divided into two groups: class I and class II [1]. Each contains large numbers of alleles, all of which bind antigen for cell surface presentation to T cells. MHC class I molecules comprise a single polymorphic chain coupled to a single conserved protein: β2 microglobulin. In contrast, MHC class II molecules comprise two polymorphic subunits: an α and a β chain. A notable difference between the classes is the open-ended peptide-binding groove of MHC class II molecules compared to the closed binding site of MHC class I. This feature, which has been observed in many X-ray crystallographic structures, has been used to explain the observed differences in peptide length accommodated by the two classes [2-4]. Class I molecules typically accommodate peptides of eight to ten residues, although instances of longer peptide binding have now been reported [5].

In contrast, MHC class II molecules can accommodate much longer peptides. Stern *et al*. investigated an influenza-derived peptide complexed with HLA-DR1; they saw the peptide bound in an extended conformation with five binding pockets, which engaged peptide side chains, while the flanking regions extended out of the groove [3]. Since MHC class II molecules have an open-ended binding groove, they do not, in general, restrict the length of bound peptides. The length of peptides occupying the groove can vary considerably as a consequence of semi-stochastic proteolytic degradation [6].

Several studies have shown the influence of residues outside the main nonameric core on binding and subsequent T cell recognition. Residues outside the nonameric binding region but within an extended binding groove have been called peptide-flanking residues (PFRs) [7]. The importance of PFRs for T cell recognition has also been investigated. It is widely accepted that these flanking regions can contribute to T cell mediated pMHC recognition [8]. Arnold and colleagues showed that certain T cell responses were completely dependent on residues at peptide positions P-1 and P11. Similar findings were reported by Stepniak *et al *for immunostimulatory HLA-DQ2 binding peptides derived from gliadin and glutenin proteins [9]. The findings of both authors can be partly explained by the results of X-ray crystallographic studies of peptide-MHC class II-TCR trimolecular complexes [10,11]. These reports indicate that the CDR3 regions of the TCR α and β chains tend to locate over the p5 residue in the binding groove allowing the TCR to interact with the PFRs. It has also been shown that the properties of residues in the PFRs can influence T cell recognition, with residues capable of forming salt-bridges or hydrogen bonding with the TCR being most favoured [8].

Much circumstantial if anecdotal evidence suggests that PFRs contribute strongly to peptide-MHC stability. They can provide an increased measurable affinity of peptide for the MHC binding groove, in particular the residue at position P-1 [12]. Nelson *et al*. reported that the effect of increased stability was greater for a peptide length increase of six amino acids compared to four. They also found the effect to differ depending on which terminus was elongated. Moreover, a previous study by Srinivasan *et al *showed increased affinity resulting from the peptide elongation of a single peptide [13]. How such peptide elongations might increase affinity remains unclear. Although it is theoretically possible PFRs interact with the MHC molecules outside the groove, this has yet to be shown experimentally, nor has it been seen in X-ray crystallographic structures.

We have sought to elucidate this phenomenon further, by examining the relationship between peptide length and MHC class II affinity. We devised custom algorithms and applied them to data available in the AntiJen database [14] to firstly ascertain whether peptide length can impact on affinity of a peptide for an MHC class II molecule. This was assessed by searching the database for instances of peptide elongation and determining the reported effect on MHC class II affinity. Following this, we examined whether there was a demonstrable limit to the effect of peptide length on MHC class II affinity and subsequently determined whether any effects on affinity were peptide terminus specific. Our analysis indicated the positive impact of peptide length on affinity. We also identified an optimal length for peptide-MHC class II binding and demonstrated that the effects on peptide affinity are terminus independent.

## Results

### Most peptide elongation events result in enhanced affinity

**Figure 1:**
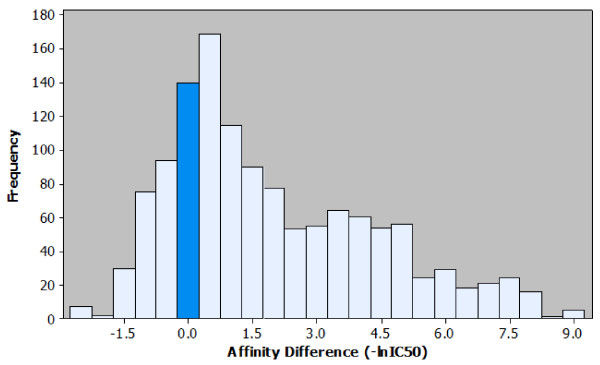
**Histogram of the affinity differences observed in the peptide elongation dataset**. Observed effects on affinity for each peptide elongation are plotted based on the frequency of occurrence. It is possible to visualise the most common affinity difference (+0.5), the most extreme affinity difference (+9.0) and the ratio of positive to negative effects. For ease of interpretation the bar representing the number of instances where no effect on affinity was observed is highlighted.

The relationship between peptide length and MHC class II affinity was examined using a dataset of peptide elongation events. This dataset comprised instances of elongation and their measured impact on MHC class II affinity for several distinct peptides and MHC alleles under normalised experimental conditions. Analysis of MHC class II binding data from the AntiJen database using bespoke algorithms generated 1279 elongation events. The dataset covered 19 distinct HLA alleles from the DR, DP and DQ loci. Most alleles were HLA-DR (Table 1). Within the dataset, DRB1*0401 was the most prominent: 25.76 percent of the characterised epitopes bound to this allele. A histogram showing the effects of peptide elongation on affinity is shown in Figure 1. Most affinity differences observed were positive. Visual examination of the affinity histogram shows an extreme value distribution with an elongated right tail, indicating a clear trend towards increased affinity in most observed binding events. However, further analysis was required to ascertain the magnitude of the effect when controlling for length of elongation and peptide length.

**Table 1:** Chi Square Table for the observed elongation events.

	**Amino**	**Both**	**Carboxy**	**All**
**Decreased (Observed)**	120	80	92	292

**Decreased (Expected)**	120.3	81.5	90.2	292

**Increased (Observed)**	407	277	303	987

**Increased (Expected)**	406.7	275.5	304.8	987

**All**	527	357	395	1279

A standard chi-square table examining bias in the observed affinity increase/decrease towards a particular peptide terminus. Rows show effect (Affinity Increase or Decrease), whereas, the columns show the terminus at which the elongation was observed.

### Peptide elongation below an optimal length results in enhanced affinity

**Figure 2:**
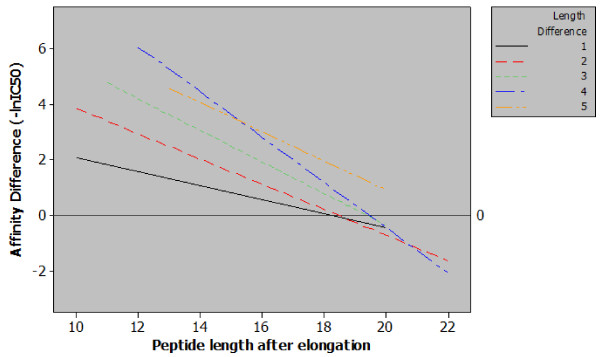
**Regression plot of classes of peptide length additions**. Five regression lines relating observed differences in affinity to final peptide length for each magnitude of peptide elongation. The content of this figure shows two important points relating sequence length and MHC class II affinity. Firstly, when correcting for final peptide length a greater length addition will generate a greater increase in affinity e.g. for peptides with a final length of 14 amino acids increasing the length by four residues has a much greater effect than a single residue increase. Secondly, from the included data it is possible to observe a length of between 18 and 20 amino acids, beyond which, increasing the length of the peptide has a null or negative effect.

The relationship between final peptide length and affinity was stratified by length of increase. A regression line was plotted for each length of increase. For reasons of data scarcity, only increases of one to five residues were analysed. Regression analysis relating the size of peptide elongations to the final length of peptides and the observed affinity indicates that a larger increase in peptide length leads to a greater gain in affinity. However, from Figure 2, it is clear that a point exists (~18–20 residues in length), beyond which there is no advantage to be gained from peptide elongation. From Figure 2, it is also clear that the positive effect on affinity due to peptide elongation lessens as peptide length increases. Examination of Figure 2 reveals that, if one were to measure the affinity of a nonameric peptide for a given MHC molecule, then serially elongating the peptide by a single residue and re-measuring the affinity, the first elongation may (on average) add 2 units to the affinity. Adding a single residue to the peptide to make it 16 residues long would add only 0.8 units to the affinity. By the time the peptide had reached 18 residues in length further elongation would decrease the affinity for the MHC molecule.

### The observed effect is terminus independent

A chi-square analysis was performed to determine if the observed effect of increased peptide-MHC class II affinity was terminus specific. Elongation events were classified by whether they resulted in an increase or a decrease in binding affinity. Events were also classified based on whether they occurred at the amino or carboxy terminus or both. A summary of the observed incidence of terminus additions and positive and negative effects on affinity are shown in Table 2. A preponderance of favourable interactions at a particular terminus might suggest that the peptide elongations at that terminus facilitated favourable interactions either inside or outside of the binding groove. The chi-square analysis indicated no significant difference between changes recorded at either termini (p = 0.959), indicating an absence of statistically significant bias. This may point to a more general, terminus independent effect on affinity.

**Table 2:** Composition of the dataset of elongation events.

**Allele**	**Count**	**Percent**
DPB1*0401	38	2.97

DPB1*0402	38	2.97

DQA1*0301/DQB1*0301	64	5

DQA1*0501/DQB1*0201	114	8.91

DRB1*0101	124	9.7

DRB1*0301	1	0.08

DRB1*0401	77	6.02

DRB1*0404	2	0.16

DRB1*0405	3	0.23

DRB1*0802	3	0.23

DRB1*0803	2	0.16

DRB1*0901	3	0.23

DRB1*1101	3	0.23

DRB1*1201	3	0.23

DRB1*1302	3	0.23

DRB1*1501	682	53.32

DRB3*0101	24	1.88

DRB4*0101	1	0.08

DRB5*0101	94	7.35

Total	N = 1279	100

The dataset of peptide elongation events contained a total of 1279 elongation events covering 19 distinct alleles. The representative numbers for each allele are preresented as raw counts and percentages.

## Discussion

While MHC class II molecules and their ligands are well characterised, the impact of peptide length on MHC affinity is not well understood. While several anecdotal observations have purported to show that peptide length may influence affinity we have for the first time systematically quantified this relationship using *in silico *analysis. Using novel computational analyses, we exploit the wealth of manually-curated quantitative binding data available within the AntiJen database [14].

When attempting to derive a relationship between sequence length and observed MHC affinity, one is limited by several factors. One such factor is the presence of different binding determinants in most sequences that could significantly bias any simple length-affinity correlation. Thus, if one examines the affinity of two sequences of different length and low sequence similarity, one cannot ascertain if an altered affinity is due to differences in the binding determinant or to changes in sequence length. To isolate such a relationship, one must normalise all factors (such as MHC allele, experimental conditions, and peptide sequence) except for sequence length before analysing the affect on affinity. To that end, we identified examples of affinity measurements, for a single sequence and an elongated equivalent, which were performed under the same experimental conditions. The benefits of this approach were twofold. Firstly, inter-laboratory variation in affinity measurements would be excluded. Secondly, bias from variations in the binding determinant would also be reduced if not eliminated, thus removing bias from intra- but not inter-sequence comparisons. Bias from inter-sequence comparisons is unavoidable. We optimised for the required task in order to avoid representing bias as effect.

The data presented in Figure 2 allowed us to examine the limits of the effect with respect to final peptide length. By examining the log fold-change in affinity for MHC class II as the length of the peptide increases it can clearly be seen that the impact on affinity lessens. The regression plot also shows that at approximately 19 amino acids the effect of peptide elongation on affinity becomes zero. Elongating peptides beyond this point would – in the majority of cases – result in a diminished affinity. This seems logical: if no such ceiling existed for the positive effect of peptide length then sequence length alone would be the key determinant of peptide-MHC affinity. This discovery is also strengthened by the findings of Lippolis *et al*. who eluted and sequenced peptides from HLA-DR*0401 and found that the most abundant species within a family of nested peptides were between 14 and 21 residues in length [15]. Our findings do not however suggest that a single optimal length would exist for all peptides for all MHC class II alleles, and indeed the relative heterogeneity of peptides binding to MHC class II molecules is well reported [15-17]. However, this approximation to an optimal length is consistent for a large number of peptide sequences and a large number of MHC class II alleles.

Many explanations for this length ceiling present themselves. It may be that as peptides become very long they are no longer able to form favourable interactions with the MHC or TCR. Alternatively, when MHC class II binding peptides attain a certain length they may form secondary or tertiary structures outside the binding site that are less compatible with MHC class II binding. An exception to this rule may be the 33-amino acid peptide initially described by Shan *et al *[18]. It binds to HLA-DQ2 and stimulates disease associated T cells. Despite its unusual length, this peptide retains its MHC class II binding abilities as its proline rich nature helps it to form a type II polyproline helix. This is the conformation normally seen in MHC class II binding grooves [3].

Our results clearly imply a general correlation between increasing MHC binding affinity and increasing peptide length. It is not peculiar to a restricted subset of peptides. Sercarz and Maverakis posited that longer peptides have a greater binding affinity for MHC class II [19]; this assertion was based on observations from a few articles, e.g. the study by Srinivasan *et al *[13]. However, to the best of our knowledge, ours is the first study to test this hypothesis systematically for several MHC class II alleles. The results of our study are compelling: across 1279 identified peptide elongation events for peptides binding to 19 distinctive MHC class II alleles, affinity predominately increased. It is important to note, however, that the examined peptides are more representative of the HLA-DR species than either HLA-DP or HLA-DQ, and this may result from an apparent publication bias in favour of HLA-DR which will be reflected in the AntiJen database.

Our findings have clear implications for experimental immunology and vaccinology, as well as computational immunology; and these implications are many. Increased affinity *in vivo *may result in increased reactivity to recently degraded peptides. Presumably, recently degraded peptides may be less completely digested and – as a result of increased length – have an increased affinity for MHC class II relative to shorter more fully degraded peptides. Such an effect would, in theory, increase the likelihood of T cells recognising more recently endocytosed peptides rather than a background of self-peptides. Additionally, the ability of exopeptidases to digest termini of peptides within the MHC class II binding cleft [6] may effectively reduce their MHC class II affinity. Thus, peptides susceptible to digestion by exopeptidases may prove to be less immunogenic *in vitro*. The field of subunit vaccine design may be able to augment vaccine efficacy by increasing peptide length or eliminating potential proteolytic cleavage sites, which may result in an inability of normal proteolytic enzymes to digest peptides to shorter equivalents with reduced immunogenicity.

## Conclusion

Using an *in silico *approach we were able to define a relationship between peptide length and MHC class II affinity. The results of this study demonstrate that, within limits, elongation of a peptide is likely to result in a greater affinity for MHC class II molecules. It was also possible to show that the optimal length for peptide-MHC affinity was approximately 18–20 amino acids. Additionally, it was also possible to demonstrate that the effects are consistent regardless of which terminus was elongated. Given the well-characterised importance of peptide-MHC interaction to the cellular immune response, it is likely that the findings of this study will prove of significance in vaccine design and the epitopic basis of immunological disease.

## Methods

### Dataset formation

Data for the study were obtained by querying the AntiJen database [14] for all sequences known to bind human MHC class II molecules. These data were imported into a spreadsheet and then further filtered to exclude those data for which no radiometrically determined IC_50 _value were present. IC_50 _values were then transformed to their -lnIC_50_. This dataset was then output to a text file containing epitope sequence, -lnIC50 value, pubmed ID of the source article, experimental conditions and the MHC binding allele. This database formed the source data for interrogation using a bespoke algorithm.

### Data query algorithm

**Figure 3:**
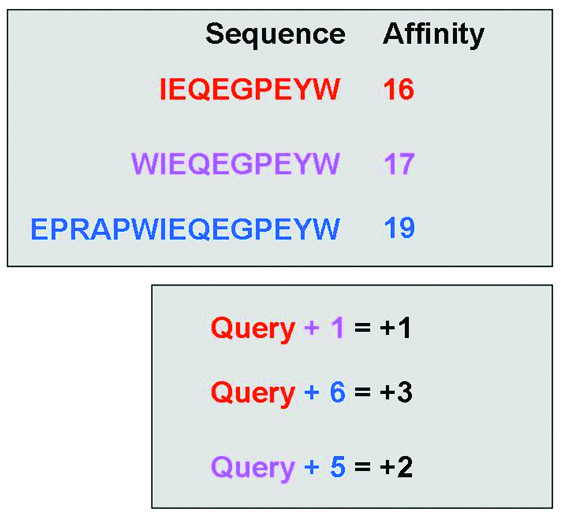
**Illustration of the elongation event search algorithm**. The upper section contains theoretical affinity data for a set of peptides within a single publication. The lower section shows the differences in peptide length and associated changes in affinity (colour coded by peptide). The algorithm searches within a publication for any instance of a sequence exhibiting a longer counterpart. The algorithm then returns the length difference and affinity difference for each recorded elongation event (i.e. Query+1 = +1, and Query +6 = +3).

To analyse the data it was decided to search for peptides for which elongation events were recorded. Different experimental conditions can give rise to variance in reported IC_50 _values for a single peptide binding to a single MHC allele. To circumvent any bias which may be introduced by comparing such peptides, it was decided to look for peptide elongation events which were performed under the same experimental conditions and published in a single article. In order to perform this search an algorithm was implemented in Python to search within a single publication for the affinity determination of a peptide and a longer counterpart. The algorithm (Figure 3) iteratively considers each peptide in order of increasing length and then searches within the publication for longer counterparts. Thus, the algorithm begins with the shortest peptides and looks for longer peptides, which represent elongated versions of the original peptide, measured under the same experimental conditions and published in the same paper. For both peptides the algorithm computes the difference in reported binding affinity and stores it in an output file with the original peptide sequence, the elongated peptide sequence and additional data such as peptide length and the Pubmed ID number of the source publication. The output file was formatted for easy entry into a statistical analysis package.

### Statistical analysis of elongation data

All statistical analyses were performed using the Minitab^® ^software package (Minitab Inc) for statistical analysis. Discrete datasets were isolated for particular analyses such as the chi-square analysis using Microsoft Excel^®^.

## Competing interests

The authors declare that they have no competing interests.

## Authors' contributions

COB developed the data sifting algorithms, performed the statistical analysis, and wrote the paper. DRF helped analyse the results and wrote the paper. CF supervised the work and wrote the paper. All authors read and approved the final manuscript.
